# The impact of transposable element activity on therapeutically relevant human stem cells

**DOI:** 10.1186/s13100-019-0151-x

**Published:** 2019-03-09

**Authors:** Gerald G. Schumann, Nina V. Fuchs, Pablo Tristán-Ramos, Attila Sebe, Zoltán Ivics, Sara R. Heras

**Affiliations:** 1 Division of Medical Biotechnology, Paul-Ehrlich-Institut, Paul-Ehrlich-Str.51-59, 63225 Langen, Germany; 2 Host-Pathogen Interactions, Paul-Ehrlich-Institut, Paul-Ehrlich-Str. 51-59, 63225 Langen, Germany; 3 GENYO. Centre for Genomics and Oncological Research, Pfizer/University of Granada/Andalusian Regional Government, PTS Granada-Avenida de la Ilustración, 114, 18016 Granada, Spain; 4 Department of Biochemistry and Molecular Biology II, Faculty of Pharmacy, University of Granada, Campus Universitario de Cartuja, 18071 Granada, Spain

**Keywords:** Adult stem cells, Genomic destabilization, LINE-1, Methylation, Pluripotent stem cells, Restriction, Regenerative medicine, Transposable elements

## Abstract

Human stem cells harbor significant potential for basic and clinical translational research as well as regenerative medicine. Currently ~ 3000 adult and ~ 30 pluripotent stem cell-based, interventional clinical trials are ongoing worldwide, and numbers are increasing continuously. Although stem cells are promising cell sources to treat a wide range of human diseases, there are also concerns regarding potential risks associated with their clinical use, including genomic instability and tumorigenesis concerns. Thus, a deeper understanding of the factors and molecular mechanisms contributing to stem cell genome stability are a prerequisite to harnessing their therapeutic potential for degenerative diseases. Chemical and physical factors are known to influence the stability of stem cell genomes, together with random mutations and Copy Number Variants (CNVs) that accumulated in cultured human stem cells. Here we review the activity of endogenous transposable elements (TEs) in human multipotent and pluripotent stem cells, and the consequences of their mobility for genomic integrity and host gene expression. We describe transcriptional and post-transcriptional mechanisms antagonizing the spread of TEs in the human genome, and highlight those that are more prevalent in multipotent and pluripotent stem cells. Notably, TEs do not only represent a source of mutations/CNVs in genomes, but are also often harnessed as tools to engineer the stem cell genome; thus, we also describe and discuss the most widely applied transposon-based tools and highlight the most relevant areas of their biomedical applications in stem cells. Taken together, this review will contribute to the assessment of the risk that endogenous TE activity and the application of genetically engineered TEs constitute for the biosafety of stem cells to be used for substitutive and regenerative cell therapies.

## Background

### Origin of stem cell types

Regenerative medicine is a recent and emerging branch of medical science, addressing functional restoration of specific tissues and/or organs of patients suffering from severe injuries or chronic diseases in a condition where the organism’s own regenerative responses do not suffice [1]. Stem cells are defined by their ability to regenerate multiple differentiated cell types, while retaining the capacity to self-replicate and self-renew. Those found in vivo have different origins and can be divided into three broad categories accordingly: embryonic (ESCs), foetal (FSCs) and adult stem cells (ASCs). Among the latter are hematopoietic stem cells (HSCs) and mesenchymal stem cells (MSCs), which represent the most used stem cell types in current clinical trials.

**ESCs** are derived from the inner cell mass of human blastocysts, have the potential for self-renewal, and are considered pluripotent: they maintain the ability to differentiate into cells and tissues from the three main germ layers [2] and generate all the tissues found in an organism. Human embryonic stem cells (hESCs) emerged as a primary cell source for regenerative medicine in recent years, to repair tissue and organ anomalies that resulted from congenital defects, disease, age and environment-associated effects. Similar characteristics are associated with induced pluripotent stem cells (iPSCs), although these are generated in vitro by ectopic expression of endogenous pluripotency factors which epigenetically transform terminally differentiated cells into ESC-like cells [3, 4].

**FSCs,** the second category of stem cells, are located in foetal tissues and embryonic annexes and are multipotent: they can differentiate into cell types from some, but not all of the three main germ layers [5]. FSCs have been subdivided into hematopoietic (blood, liver, bone marrow), mesenchymal (blood, liver, bone marrow, lung, kidney and pancreas), endothelial (bone marrow, placenta), epithelial (liver, pancreas) and neuronal stem cells (neurons, glia and oligodendrocytes [6].

The third category, termed ASCs or progenitor cells, comprises multipotent tissue-resident stem cells found in fully developed tissues. They reside in niches that create a special microenvironment for their replication and self-renewal. Domiciled in most tissues of the human body, discrete populations of ASCs generate cells to replace those that are lost through normal repair, disease, or injury. ASCs are found throughout the lifetime of the organism and were identified in tissues such as the umbilical cord, placenta, bone marrow, muscle, brain, fat tissue, skin, gut, etc. ASCs are thought to act to repair and regenerate tissues in which they reside, helping to maintain tissue homeostasis.

### Clinical application of adult and pluripotent stem cells

**Fig. 1:**
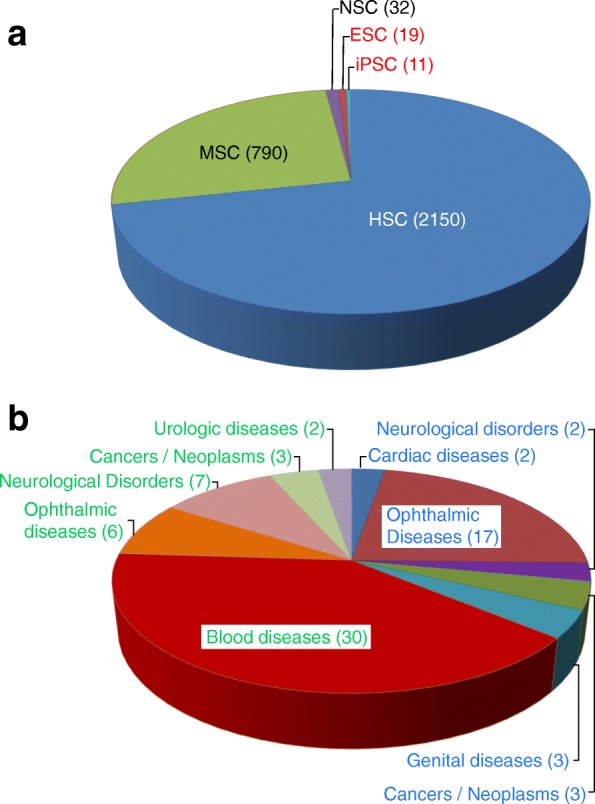
Human stem cell-based clinical studies conducted worldwide (Status: October 2018; www.clinicaltrials.gov). **a** Interventional clinical trials (Phase I or phase I/II) applying adult stem cell types (mesenchymal stem cells, MSCs; hematopoietic stem cells, HSCs; neuronal stem cells, NSCs) or their differentiated derivatives, or embryonic stem cell (ESC)-or induced pluripotent stem cell (iPSC)-derived differentiated cells. Numbers in brackets indicate the number of individual trials based on the respective stem cell type. **b** Interventional clinical trials and observational studies that are currently ongoing or in preparation use therapeutic derivatives of ESCs (blue lettering) or iPSCs (green lettering) to treat ophthalmic, urological, blood, cardiac and genital diseases, neurological disorders and cancers/neoplasms. Numbers in brackets represent the number of clinical trials and/or observational studies initiated to treat the respective disease or disorder

**Adult stem cells** such as HSCs, MSCs, and neural stem cells (NSCs) are the most frequently used cell types in interventional clinical trials (www.clinicaltrials.gov) (Fig. 1a). HSCs are essential for the generation and homeostasis of the blood system, and give rise to all blood cell types, including lymphocytes, erythrocytes, monocytes, granulocytes, and platelets [7]. Indeed, HSC transplantation is the accepted therapy of choice for a variety of malignant and non-malignant blood-related diseases in children and adults. Initially developed as rescue therapy for a patient with cancer after high doses of chemotherapy and radiation, as well as the correction of severe deficiencies in the hematopoietic system, it has evolved into an adoptive immune therapy for malignancies and autoimmune disorders [8]. Human MSCs are multipotent stem cells with the capacity to differentiate into the mesodermal lineage such as osteocytes, adipocytes and chondrocytes, as well as some ectodermal (neurocytes) and endodermal lineages (hepatocytes) [9]. MSCs have been isolated from various tissues including bone marrow, adipose tissue, amniotic fluid, endometrium, dental tissues, umbilical cord and Wharton’s jelly [9]. MSCs have immunomodulatory features and secrete cytokines and immune receptors which regulate the microenvironment in the host tissue [10]. Multilineage potential, immunomodulation and secretion of anti-inflammatory molecules makes MSCs an effective tool in the treatment of chronic diseases. Consequently, there are several clinical trials harnessing their innate characteristics or other in vitro observations in a series of diseases (for a review see [11]). NSCs are a group of ectodermal progenitor cells which can differentiate into neural subtypes, such as neurons, astrocytes or oligodendrocytes. Neural stem cell derivatives are used in a number of clinical trial applications including treatments of ALS and Parkinson’s Disease [12–14] and currently ongoing interventional clinical trials (Fig. 1a).

In contrast to MSCs and other types of tissue-specific stem cells, pluripotent stem cells (PSCs) are derived either from human pre-implantation embryos, giving rise to hESCs [2, 15], or from somatic cells that are reprogrammed to a primitive pluripotent state (hiPSCs). PSCs are immortal and highly expandable in culture in vitro*,* and can be differentiated to almost any cell type of the body. Their potential for regenerative medicine is therefore unique and extraordinary. Indeed, cellular products derived from hESCs are now in clinical trials for cardiac and ophthalmic diseases and neurological disorders, with some other applications registered for clinical trial approval (Fig. 1b) [12–14]. Initially, hiPSCs have been used in one experimental procedure in an autologous approach on an individual in Japan with macular degeneration [16, 17]. In March 2017, the first study was initiated involving 5 AMD (Age-related macular degeneration) patients who received retina cells derived from banked hiPSCs in an allogeneic approach [18]. To date, 11 interventional clinical trials and 25 observational studies are based on the application of iPSCs (Fig. 1). However, and despite these trials in the frontier of knowledge, relatively little is known about undesired long-term effects of such approaches.

### The issue of genomic integrity

The promise for human disease treatment using differentiated cells derived from multipotent ASCs and pluripotent stem cells, such as hESCs and hiPSCs, also carries the threat of genomic instability of the cells to be administered. Firstly, cultivation of multipotent and pluripotent stem cells exposes the cells to selection pressures that often result in the acquisition and manifestation of genomic alterations, varying in size from point mutations, through copy number changes in small genomic elements (e.g. amplification of repetitive sequences and retroelement mobility), to large chromosomal aberrations, trisomies and monosomies [19–21]. Previous reviews reported several factors that contribute to differences in genomic and epigenomic stabilities of stem cells, including derivation source (embryonic vs. somatic cells), derivation methods (direct isolation vs. reprogramming), and culture conditions [22]. Much attention has been drawn in recent years to the genomic aberrations acquired by hESCs and hiPSCs, ranging from point mutations to whole-chromosome trisomies [23–30]. Similarly, human ASCs that are expanded in culture were also shown to be prone to acquire chromosomal aberrations [24]. Secondly, the treatment of many human diseases often involve genetic manipulation of stem cells prior to transplantation, which may further jeopardize their genomic stability. Overall, genomic aberrations can affect identity, differentiation capability and tumorigenicity of stem cells, and should thus be routinely evaluated for their proper use in basic research and in clinical trials. In the promising era of stem cell research and therapy, ensuring genomic stability of stem cells and their derivatives remains one of the highest priorities prior to clinical translation.

In this review, we focus on one specific source of genomic instability in human therapeutically relevant stem cells that has been mostly ignored by the stem cell community to date, namely the activity of endogenous non-Long Terminal Repeat (non-LTR)-retrotransposons, and the consequences for genomic integrity and host gene expression. Non-LTR retrotransposons constitute our center of attention because in contrast to most TEs in our genome, a small fraction of this group of TEs is currently active and mobilized in the human population [31, 32]. We provide an overview of the impact of endogenous TEs in pluripotent and adult stem cells, discuss new roles of TEs in regulating pluripotency, and describe host defense systems counteracting TE activity in stem cells. Furthermore, we address the application of DNA-transposons to genetically engineer human stem cells for medical applications. In order to fulfill the standards for safe clinical applications and evaluate the risk for biosafety inflicted on therapeutically relevant cells by TE activity, we propose that the extent of the activity of potentially mutagenic TEs in pluripotent stem cells needs to be elucidated, and perhaps used as an additional quality control check point in the future.

## Non–LTR retrotransposons are currently active in the human genome

### Retroelement families

**Fig. 2:**
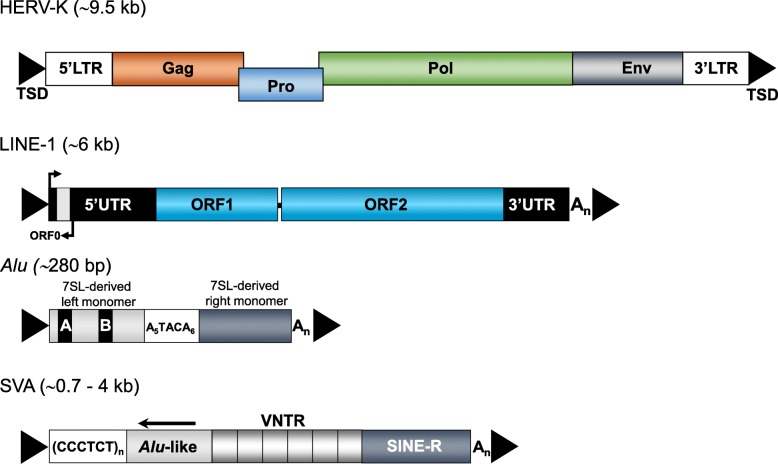
Retrotransposons in the human genome. Currently, only LINE-1 (L1), *Alu* and SVA elements are verifiably still mobilized in humans**.** A full length HERV-K provirus is ~ 9.5 kb long, codes for group-specific antigen (Gag), protease (Pro), polymerase (Pol) and envelope (Env) proteins and is flanked by ~ 1-kb long terminal repeats (LTRs) with the 5’LTR including the HERV-K promoter. A functional full length L1 element is ~ 6 kb in length, harbours three open reading frames (ORF0, ORF1 and ORF2) at which ORF1 and ORF2 are separated by a 63-bp noncoding spacer region. The 5′ untranslated region (5’UTR) harbours both sense and antisense promoter. *Alu* elements comprise ~ 280-300 bp, are composed of two 7SL-RNA derived monomers separated by an A-rich connector (A_5_TACA_6_), contain an internal A and B box promoter, and end in a poly A tail (A_n_). SVA elements are ~ 0.7–4 kb long, consist of a 5′ hexamer repeat that can be variable in length ((CCCTCT)n), two *Alu* fragments in antisense orientation, a GC-rich variable number of tandem repeats (VNTR) region, a SINE-R sequence derived from an HERV-K10 element and a poly A tail following a polyadenylation signal. The length of an intact SVA can vary depending on the number of repeats present in the hexamer and VNTR domains. L1, *Alu* and SVA insertions are characterized by the hallmarks of L1-mediated retrotransposition such as flanking variable target site duplications (TSDs), polyA tails at their 3′ ends (A_n_) and insertion at the consensus target sequence 5′-TTTT/AA-3′. 3’UTR, 3′ untranslated region

More than 45% of the human genome is made of TE-derived sequences, but only a specific subset of these TEs that belongs to the group of retrotransposons is currently propagating in the human genome. Retrotransposons spread by a ‘copy-and-paste’ mechanism that involves reverse transcription and insertion of an intermediate RNA at a new site in the genome. They are classified in two different groups: LTR-retrotransposons, which include human endogenous retroviruses (HERVs) and are flanked by long terminal repeats harbouring transcriptional regulatory domains, and non-LTR retrotransposons that lack LTRs (Fig. 2). Among the 31 human endogenous retrovirus subfamilies covering 8% of the human genome, replication-competent HERVs have not been described to date, although their existence cannot be ruled out [33, 34]. The only TEs that are currently verifiably mobilized in the human genome are members of the group of non-LTR retrotransposons (Fig. 2). They comprise two main types of elements: i) autonomous Long interspersed class 1 elements (LINE-1s or L1s), and ii) non-autonomous short interspersed elements (SINEs) such as *Alu* and SVA (SINE-VNTR [variable number of tandem repeats] -*Alu* elements) elements. In humans, > 500,000 L1 copies occupy ~ 17% of the haploid genome [35]. A functional, full-length L1 is 6 kb in length and initiates transcription using an internal sense-promoter located within its 5′ untranslated region (UTR) [36–38] (Fig. 2). In functional L1s the sense promoter drives expression of two proteins, named ORF1p and ORF2p, that catalyze L1 retrotransposition in *cis* [39]. ORF1p is a 40 kDa RNA binding protein with nucleic acid chaperone activity, while ORF2p is a 150 kDa protein that contains reverse transcriptase (RT) and endonuclease activities (reviewed in [32]). Both proteins are strictly required for retrotransposition [40] by a process termed Target-Primed Reverse Transcription (TPRT; [41, 42]). A conserved antisense-promoter in the L1 5’UTR [36, 37] drives expression of the *trans*-acting polypeptide ORF0, that can stimulate L1 mobilization [43]. Although the RT activity of human L1 was shown to be highly processive [44], most de novo L1 insertions are rendered immobile by 5′ truncation. These truncations together with internal mutations accumulated over time, have resulted in only 80–100 retrotransposition-competent or functional L1s per individual human genome [45–47]. Of these, fewer than 10 mobilize efficiently when tested in vitro: they are termed ‘hot’ L1s and responsible for the bulk of retrotransposition in the human population [45, 47–50]. The vast majority of hot L1s belong to the youngest subfamily, termed L1Hs (for Homo sapiens-specific, also known as L1-Ta, for transcribed-active) [45, 47, 51]. L1 subfamilies can be distinguished by nucleotide substitutions that are shared by all members of a specific subfamily. Five subfamilies are thought to have amplified in hominoid primates within the past 25 myrs, named L1PA1 to L1PA5. Functional L1 s are also responsible for the mobilization of *Alu* and SVA elements which lack protein-coding capacity [52–55]. Indeed, L1 activity *in trans* can act at low frequency on any cellular polyadenylated mRNA, resulting in the generation of processed pseudogenes [56, 57]. Members of the primate-specific *Alu* family are approximately 300-bp long, have a dimeric structure, and are transcribed by RNA polymerase III (reviewed in [58, 59]) (Fig. 2). The family of *Alu* elements is, with > 1 million copies, the most abundant TE in our genome [35], and represents the currently most active group of retrotransposons in humans with ~ one thousand mobilization-competent copies per genome. The SVAs are hominid-specific composite non-coding retroelements, with an average length of ~ 2 kb, and represent the youngest family of active human TEs (Fig. 2). There are roughly 2700 SVA copies per human genome, most of which are full-length and about 20–50 of which may be active [60–67].

In sum, all above listed mobilization-competent non-LTR retrotransposons may collectively account for thousands of somatic insertions [68]. It was calculated that up to 5% of newborns harbour a new retrotransposition event, and > 120 known human disease-causing insertions of L1 s, *Alu*s and SVAs have been identified to date [69–73]. L1-mediated insertions have sporadically resulted in a wide number of human genetic disorders, including hemophilia A (L1), cystic fibrosis (*Alu*) [74] and X-linked dystonia-parkinsonism (SVA) to cite a few reviewed in [71]. Altered expression of TEs and animation of genomic L1 sequences also appear to be hallmarks of cancer, and can be responsible for driving mutations in tumorigenesis [75, 76]. Indeed, the random mobilization of L1 in our genome implies that virtually any disorder can be generated by the mutagenic activity of non-LTR retrotransposons, and L1-mediated retrotransposition events occur approximately once in every 250 pathogenic human mutations [77].

### TE insertions can affect host gene expression by various mechanisms and impact stem cell development

When expressed, TEs can affect developmental processes either by their encoded gene products, which could influence phenotype and/or function of the host cell, or through their genomic de novo insertion, that could result in genetic changes in the host genome. L1-mediated retrotransposition events can be associated with the induction of local genomic instability, including DNA deletions at the insertion site, 3′- or 5′-transductions, and non-allelic homologous recombination (reviewed in [32, 78]). Additionally, new retrotransposon insertions into genes can act as insertional mutagens, alter gene expression and interfere with host gene function [79].

Although fewer than 1 of 10,000 TEs is still capable of mobilization [71], a far greater proportion can influence gene expression in *cis* and in *trans* (reviewed in [80]). This is because TEs, such as ERVs, L1 and SVA elements, contain transcription factor binding sites that promote transcription by RNA polymerase II or, in the case of *Alu* elements, by RNA polymerase III. Therefore, the promoter(s) of resident and new TE(s) that integrated into or close to host genes, can alter gene expression, and even lead to the appearance of novel transcripts that encompass part of the coding region. For instance, because transcripts of several host genes in iPSCs originate from a nearby L1 antisense promoter, the most abundant transcript isoforms expressed contain L1 ORF0 sequences whereas for other genes, these isoforms contribute only little to the bulk transcripts. Interestingly, ORF0 transcript levels were dramatically downregulated in the source fibroblasts relative to their derived iPSCs [43]. De novo genic insertions can also induce premature termination of transcription by introducing TE-derived polyadenylation signals [81]*,* and sporadically result in the exonization of TEs. Exonized TEs can expand the mammalian transcriptome and proteome, but can also be used to fine-tune gene regulation [82]. Indeed, nucleotide sequences of several classes of TEs have been found inserted within mammalian RNAs [82–84] raising the possibility that they affect gene regulation and function by providing or interfering with regulatory elements in those RNAs. While the decrease in host gene RNA expression due to inadequate transcript elongation after L1 insertion in an intron of a transcribed gene was originally attributed to the A/T richness of the L1 sequence [85], it was suggested recently that transcriptional attenuation of host gene expression could be a consequence of epigenetic silencing mediated by the human silencing hub (HUSH) complex [86]. TE insertions can also introduce TE-derived splice acceptor or donor sites that alter splicing, and thereby generate non-functional or nonsense transcripts [87] or can be incorporated into mRNAs and thus introduce frameshifts or premature termination codons. It has also been shown recently that spliced L1 mRNAs are also used as templates generating spliced integrated retrotransposed elements (SpIREs) which lack part of the 5’UTR and, consequently, lack some of the regulatory sequences [88]. L1-encoded proteins can sporadically generate processed pseudogenes [39, 56], and thereby affect the stem cell transcriptome and proteome. Finally, ongoing L1 retrotransposition itself can copy regulatory sequences such as promoter and enhancer regions and/or protein coding regions and introduce them into new sites in the host genome by a mechanism termed “exon shuffling” [89]. Exon shuffling occurs when an L1 insert resides within a gene, L1 transcription bypasses the inherent weak L1 polyadenylation signal, and transcribes downstream exons generating a chimeric RNA, which could then retrotranspose to a new genomic location. It is estimated that ~ 15–20% of the L1Ta subfamily members in the human genome contain 3′-transduced sequences. Such L1-mediated 3′ transductions are responsible for as much as 1% of the human genome [90, 91], and this process has probably increased the repertoire of the human proteome. In summary, there are multiple manners in which TE insertions can impact the functioning and regulation of the human genome.

## Activity of non-LTR retrotransposons in human pluripotent stem cells

### Primary milieu for the evolutionary struggle between TEs and the host genome are pluripotent cells of the early mammalian embryo and germ cells

**Fig. 3:**
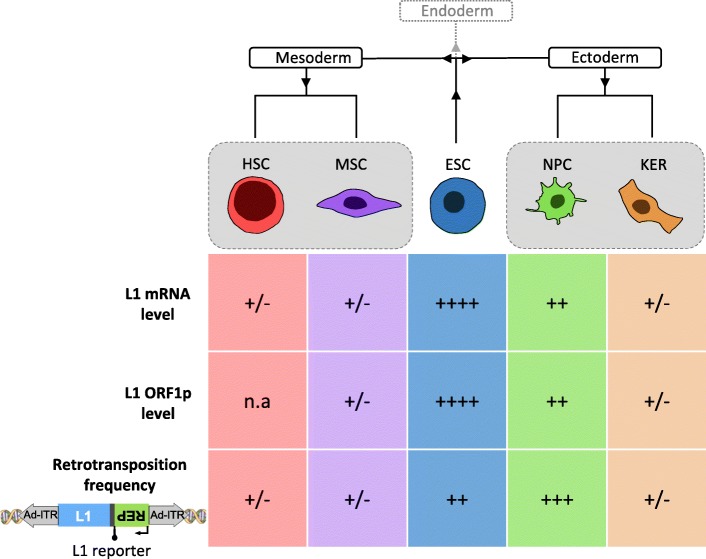
Schematic of relative endogenous L1 expression levels and engineered retrotransposition frequencies in human ESCs and adult stem cells derived from mesoderm and ectoderm. Relative L1 mRNA and L1 ORF1p expression levels, and relative engineered L1 retrotransposition frequencies supported by the respective cell type are illustrated: ++++, very high; +++, high; ++, moderate; +/−, barely detectable and therefore very low. HSC, Hematopoietic Stem Cells; MSC, Mesenchymal Stem Cells; ESC, Embryonic Stem Cells; NPC, Neural Progenitor Cells; KER, Human Foreskin Keratinocytes

To ensure their evolutionary success, TEs need to mobilize in germ cells and/ or during early development, before germline partitioning, to guarantee transmission of new retrotransposition events to the next generation. A genome-wide epigenetic switch in the early mammalian embryo, characterized by global DNA demethylation, is necessary to activate the programme of embryonic development. This ‘epigenomic reset’ is thought to provide a window of opportunity for retrotransposons to mobilize and create heritable insertions [92], as L1 expression is also controlled by DNA methylation [93]. Consistently, previous studies have shown that i) L1 mRNAs are present in human immature diploid oocytes from in vitro fertilization donors [94], ii) both L1-encoded proteins, ORF1p and ORF2p, are expressed in prespermatogonia of fetal testes and in germ cells of adult testes [95], and iii) hESCs overexpress a combination of potentially functional full-length L1 and core *Alu* elements as well as non-functional L1 and *Alu* RNAs (Fig. 3) [79, 96–100]. The observed L1 expression levels in these pluripotent stem cells inversely correlate with both their low global DNA methylation levels, and their levels of L1 promoter methylation [97, 101, 102]. It has been reported that only a specific subset of L1 retrotransposons, likely under the combined influence of activators or repressors and local chromatin constraints, is active at any given period of early embryogenic development type [67, 96, 103, 104]. While expression of members of the youngest L1 lineages, L1Hs and L1PA2, is highest in hESCs, expression of a subset of L1 lineages, primarily L1PA3, L1PA4, L1PA5 and L1PA6, which were predicted to have entered the ancestral genome between 26.8 and 7.6 million years ago, is reduced [98]. Thus, these data demonstrated that RNAs from active and “hot” L1 s are expressed in hESCs and hiPSCs. Using an engineered L1 retrotransposition reporter assay in cultured cells [40], it was demonstrated that the cellular environment of human and murine ESCs support L1 retrotransposition (Fig. 3), and that L1 de novo insertions in cultivated hESCs can occur into genes and lead to small deletions of genomic DNA at the target site [97, 105, 106]. In agreement with these data demonstrating that host factors essential for retrotransposition of functional L1 mRNAs are present in hESCs, it was verified that endogenous *Alu*Ya5 elements are *trans*-mobilized in cultured hiPSCs [79].

### Somatic and heritable L1-mediated retrotransposition in pluripotent stem cells during early development

Studies of transgenic mouse models established to investigate engineered L1 retrotransposition in vivo*,* and, more recently, the sequencing of mouse pedigrees, confirmed that the majority of heritable de novo retrotransposition events in mice occur in preimplantation embryos, and indicate that embryonic cells are likely to be the natural habitat for L1-mediated retrotransposition [107–113]). Notably, such L1 mobilization in pluripotent cells of pre-implantation embryos could result in the new insertion being a mosaic within somatic and germline cells in adult mice. Although it was reported that L1 transcript levels peak after fertilization at the 2-cell-stage and decrease from the 2- to the 8-cell stages in mice [114], it remains unclear at which time point after fertilization L1-retrotransposition is initiated. Endogenous L1 elements were shown to be active in mouse early primordial germ cells, before the PIWI/piRNA retrotransposon defense pathway is activated in male embryonic gonads [113, 115].

By contrast, we currently know relatively little about TE expression and timing of retrotransposition in humans. An example that unambiguously proves that endogenous L1 mobilization can occur early in human embryogenesis was reported by van den Hurk and colleagues: they characterized a mutagenic L1 insertion resulting in choroideremia, a rare recessive X-linked eye disorder, in an affected male patient whose mother was a somatic and germline mosaic for the pathogenic L1 insertion [116]. Due to a 3′ transduction carried by the mutagenic L1 insertion, the authors were able to identify the specific donor L1 element and verify that it was retrotransposition-competent [116, 117]. Taken together, data strongly suggest that, in humans, new heritable L1 and *Alu* insertions can likely accumulate during early embryogenesis, and to some extent in germ cells [97, 107, 110]. Retrotransposition events mediated by L1 were estimated to occur, at a minimum, in 1 of 20 meioses for *Alu*, 1 of 20 to 200 meioses for L1, and 1 of 900 meioses for SVA [118]. Clearly, future studies should determine the timing of L1 retrotransposition during embryogenesis and a rate of retrotransposition in modern humans.

Very recently, studies in mice provided evidence that the observed transcriptional L1 activation may have a role in mouse ESCs and pre-implantation embryos. Activation of L1 transcription after fertilization regulates global chromatin accessibility at the beginning of development, and therefore could be important for normal embryonic development [119]. Also, the L1 RNA seems to act as a nuclear scaffold to repress the transcriptional program specific to the 2-cell embryo and, consequently, the L1 transcript could be required to exit from the 2-cell stage [120]. These data suggest that L1-encoded RNAs and proteins have additional roles in embryonic development. These findings seem paradoxical, as despite the mutagenic potential of L1 during embryogenesis, they suggest that L1 activity could be required for proper embryonic development and even to maintain ESC identity.

### TE activity as parameter to define the naïve pluripotent state in hESCs

Mouse ESCs and iPSCs were proposed to represent a naïve state of pluripotency corresponding to the inner cell mass, whereas hESCs/iPSCs correspond to a more advanced, or ‘primed’, state of pluripotency found in the postimplantation epiblast [121]. Indeed, it has been challenging to define the naïve state of pluripotency in hESCs, particularly in view of the expanding number of protocols for deriving putative naïve human cells [122, 123]. Putative naïve human cultured cells were examined according to their resemblance to human pluripotent cells in vivo. Three comparative parameters for evaluating the naïve state of human pluripotent stem cells were established: (1) the expression profile of TEs based on single-cell RNA-seq data [124], (2) the DNA methylation landscape of human preimplantation development [125], and (3) the chromosome X inactivation status of female human ESCs [126, 127]. Comprehensive examination of the “transposcriptome” was found to offer a more sensitive measure of the correspondence between pluripotent stem cells and the early human embryo than gene expression profiling [128]. Constituting a unique model system to dissect mechanisms of early human development, naïve hESCs provide an excellent cellular model to interrogate the function of transposable elements that become activated during early human development [128].

### Cultured human pluripotent stem cells support expression and mobilization of endogenous TEs

Human iPSCs are currently successfully applied for disease modeling, the study of cell development and function, and for in vitro screening of drug candidates on healthy and diseased cells; thus, cellular products derived from hiPSCs hold substantial promise for substitutive and regenerative, autologous and allogenic cell therapies. Unlike hESCs, hiPSCs are a potential source of autologous cells compatible with the immune system of transplant recipients [129]. hiPSCs also circumvent ethical issues associated with the use of human embryos to derivate hESCs [129]. However, genetic and epigenetic aberrations that occur during reprogramming and in vitro expansion [25–28, 79, 130] may hinder the use of hiPSCs in regenerative medicine because they could affect their identity and differentiation capability, and could elevate the risk of tumorigenesis upon implantation [131]. Thus, identifying the full spectrum of aberrant mutational processes accumulated in hiPSCs, and their functional consequences, is of paramount significance. A direct consequence of reprogramming, due to genomewide epigenomic changes [130], is the activation of full-length L1 mRNA expression; indeed, L1-ORF1p and L1 RNA transcript levels are upregulated by up to four orders of magnitude compared to their respective parental cells [79, 100, 132, 133]. To investigate if hiPSCs provide the environment for L1 retrotransposition, Wissing and colleagues exploited an in vitro L1 retrotransposition assay, and detected similar levels of L1-engineered retrotransposition as those previously reported in hESCs [97, 100]. These data demonstrated that the cellular milieu of both hESCs and hiPSCs contains the host factors required for L1 retrotransposition. In order to clarify if the transcriptional activation of endogenous L1 elements in hiPSCs leads to L1-mediated mobilization, targeted sequencing was performed on cultured hESC and hiPSC lines, followed by PCR validation of candidate de novo L1, *Alu* and SVA insertions in multiple laboratories [79]. Notably, the sequencing of a small number of lines revealed ongoing L1, *Alu* and SVA retrotransposition in hiPSCs; Similarly, one *Alu* de novo insertion was found to have occurred during the cultivation of the hESC line H9, which was the only hESC line analyzed in this study [79]. Thus, these studies confirmed that hESCs and hiPSCs are a natural habitat for L1-mediated retrotransposition, consistent with heritable retrotransposition during early human embryogenesis. However, the currently available datasets on L1 mobilization in hiPSCs vs hESCs are not sufficient to allow conclusions on the frequency of L1-mediated retrotransposition events in individual hESCs or hiPSCs. Remarkably, an earlier whole-genome sequencing (WGS)-based study in mouse ESCs failed to detect endogenous L1 retrotransposition [134]. Lack of retrotransposition in mESCs when compared to hESCs/hiPSCs might reflect distinctly different properties of human primed [79] and mouse naïve [134] pluripotency states; or the limitations of the method employed for detecting de novo retrotransposition events, namely genome-wide DNA sequencing with [79] or without [134] a previous retrotransposon enrichment step. Overall, these data might suggest that the genome of mESCs would be more stable than hESCs/hiPSCs, although several studies have presented compelling evidence demonstrating ongoing (and heritable) L1 retrotransposition during early mouse embryogenesis [107, 113]. Thus, it is also possible that cultured mESCs fail to recapitulate this aspect of mouse early embryonic biology, and additional studies are required to solve this contradiction.

### Occurrence and structures of de novo retrotransposition events in cultured hESCs and hiPSCs

An intriguing aspect of L1 retrotransposition studies in hESCs/hiPSCs is that most validated insertions are full-length. In hiPSCs, 57–66% of all validated endogenous and engineered L1 de novo retrotransposition events were full-length [79, 100]. Another report found seven potential de novo L1 insertions in two hiPSC lines using targeted L1 sequencing, but could not PCR-validate or fully characterize the genomic integration sites of these events [133]. The success in the identification of L1 mobilization events depends on multiple factors such as heterogeneity of the investigated cell population, and methodological factors such as stem cell culture conditions, population bottlenecks in cultured cells, high-throughput sequencing method used, bioinformatic parameters, and how candidate L1 insertions are validated, which can affect the results decisively. For example, a recent study applied WGS to nine hiPSC lines [135] and did not identify any de novo retrotransposon insertions, and far fewer mutations overall when compared to earlier studies [25, 26]. While an early report in hESCs validated a small number of engineered but 5′-truncated L1 insertions [97], additional insertion characterization in hESCs has recently revealed that > 70% of them are full-length (Cano and Garcia-Perez, personal communication). The finding that 66–75% of engineered L1 retrotranposition events in hESCs and hiPSCs are full-length is in stark contrast to the situation in cancer cell lines, where only ~ 5% of engineered L1 de novo insertions represent full-length elements [136]. While this discrepancy warrants additional research, it is tempting to speculate that due to L1 overexpression, host factors involved in 5′ truncation might be titrated/sequestered in hESCs/hiPSCs allowing a higher rate of full-length insertions in these cells. Notably, other features of de novo L1 insertions events do not differ significantly between cancer cells and hESCs/hiPSCs; For example, ~ 50% of all validated L1 retrotransposition events in both cell types occurred into introns of host genes [79, 100, 136]. Similar to transformed cells, intronic L1 insertions into hiPSCs can interfere with the transcription of the affected host gene [79].

### Epigenomic reprogramming causes a constant derepression of endogenous TEs

Despite the unknown aspects of L1 biology in ESCs, available data indicate that reprogramming elicits a dynamic but consistent derepression of endogenous L1 s, and that they experience a relaxation of host genome control in pluripotent stem cells obtained from embryonic tissues [111, 128, 132, 137, 138]. A recent study examining transcriptional control of endogenous retroelements during the reprogramming of human hematopoietic stem cells from cord blood and primary hepatocytes into hiPSCs and subsequent cultivation uncovered a marked transcriptional upregulation of active SVA_A and L1Hs retrotransposons, but also of currently inactive HERV-K, HERV-H and HERV-W elements [132]. A sharp increase of L1 and SVA transcription was observed several days after transduction with reprogramming vectors, and both RNAs remained highly expressed upon culturing hiPSCs [132]. While there was only little heterogeneity in L1 expression levels between tested hESC lines, considerable heterogeneity between hiPSC clones was observed [132]. Comparison of expression levels of individual endogenous retroelements between hiPSC clones derived from a single donor and issued from the same reprogramming experiment uncovered striking differences, notably for HERV-H, HERV-K and L1Hs. Thus, these data suggest that the genome wide epigenomic reprogramming might not occur to the same extent in all reprogrammed cells, and that hESCs might represent a more realistic model to study human early embryogenesis processes. It was hypothesized that transcriptionally activated functional L1Hs elements, whose antisense promoters can affect the expression of neighbouring genes [139], have a similar effect during or after reprogramming of hiPSCs [132]. The interference of *cis*- and *trans*-acting transcriptional regulatory elements of preexisting or de novo TE insertions with the transcription of TE-close genes may result in phenotypic anomalies difficult to detect through conventional assays, such as blockade of differentiation to particular lineages, predisposition to oncogenic changes, aberrant release of bioactive molecules, altered immunogenicity or ectopic activation of disease-related genes in iPSCs or their progeny [67, 132]. In hiPSCs, unsteady production of transcripts from TE-integrants normally silenced in ESCs and secondary activation of neighbouring genes was noted [67]. This phenomenon could contribute to the inefficiency of reprogramming by stochastically activated genes that affect the path to pluripotency [140]. Transcriptional perturbation of TE-close genes may also plague iPSCs and their progeny with phenotypic anomalies [141]. These findings argue for an in-depth survey of the genomic, transcriptional, and epigenetic state of the repetitive genome of iPSC clones, that are to be used for basic research or clinical applications. In these studies, bona fide established hESC lines could offer an excellent reference to detect harmful changes in hiPSCs that might limit their applications. Indeed, and while the use of hESCs on regenerative medicine is clearly limited, hESCs have become an excellent benchmark to establish clinically safer human pluripotent embryonic stem cell-like cells.

## Activity of endogenous non-LTR retrotransposons in adult stem cells

### Breaking the dogma – TE activity in adult stem cells of the brain

TEs are the prototype of “selfish DNA”, whose only purpose is to generate more copies of themselves. Thus, it was assumed for a long time that most TE activity in mammals might occur only in cellular niches that transmit genetic information to the next generation, as this would ensure the evolutionary success of TEs. Although pioneer work by Barbara McClintock discovered TEs in somatic tissues of corn [142], the lack of TE activity in the mammalian soma has been a long-standing dogma. However, this “dogma” was recently challenged with the description of TE activity in mammalian healthy somatic cells, specifically in adult stem cells of the brain. The first evidence for ongoing mobility of L1 s in adult stem cells was obtained from neural precursor cells (NPCs) derived from rat hippocampus neural stem cells [112]. It was demonstrated that rodent NPCs can support elevated levels of retrotransposition using engineered human L1 reporter elements. By applying a transgenic mouse model it was demonstrated that L1 s generate genomic somatic mosaicism during brain development and in adult mice. Consistently, human NPCs isolated from fetal brain or derived from hESCs were shown to express moderate levels of L1 RNA and L1-ORF1p (Fig. 3), and to significantly support elevated levels of engineered L1 retrotransposition in vitro [143]. These and previous studies in mice (i.e., [112]) represented a major breakthrough in the TE field, because it was demonstrated for the first time that the endogenous L1 copy number is increased in several regions of the human brain (including hippocampus) when compared to other human somatic tissues [143, 144]. More recently, the use of an adenoviral/L1 hybrid vector [145] has allowed demonstrating L1 retrotransposition in non-dividing mature neuronal cells, differentiated from hESCs [99] (Fig. 3). While L1 s are usually repressed in somatic tissues, the mechanism responsible for L1 activation in neuronal progenitor cells has been studied (for review see [146, 147]). While the L1 promoter whose activity is known to be regulated by CpG methylation [93, 148], and is hypermethylated in mature neurons, it is less methylated than in fibroblasts [143] and these data suggest that additional factors beside DNA methylation might allow L1 to be expressed in neurons. The most accepted current model for the regulation of L1 expression and retrotransposition in neurons and NPCs involves the activity of Sox2 and WNT3A in combination with DNA methylation. Briefly, Sox2, a negative regulator of neuronal differentiation, is suggested to interact with the L1 promoter and repress L1 expression in rodent and human neural stem cells [112, 143]. Methyl-CpG-binding protein 2 (MeCP2) has been demonstrated to associate with the L1 promoter in a methylation-dependent manner, and to repress L1 expression in neural stem cells [149, 150]. Upon differentiation, Sox2 is downregulated, the promoter of L1 is mildly demethylated and thereby decreases binding of MeCP2 and increases L1 expression. At the same time, activation of WNT3A could stimulate L1 expression through the canonical Wnt pathway [151].

### Somatic L1 retrotransposition events accumulate during various developmental steps

Above studies mostly used engineered L1 reporter constructs, although more recently sequencing-based approaches have been used to demonstrate ongoing endogenous L1 retrotransposition in the human brain, not only in NPCs but also in neurons [144, 152–154]. Several high-throughput sequencing methods for the detection of endogenous retrotransposition events have been recently developed and applied to brain-derived samples (reviewed in [155]). Retrotransposon capture sequencing (RC-seq, [156]), and L1-seq [72] are two of the methods used to demonstrate L1 retrotransposition in neuronal cells isolated from cerebral cortex, caudate nucleus and hippocampus. Although these studies have reported significant variability in the inferred rate of L1 mobilization in the brain [144, 152–154, 157], the main conclusion from these studies is that somatic L1 retrotransposition events are accumulated during a variety of neural development stages, including early progenitor cells and mature neuronal cells. In summary, and while current estimates of L1 retrotransposition rates in neurons range from 0.04–0.6 to 13.7 L1 insertions per cell (see [111, 146, 157]), it is clear now that our brain is composed of a mosaic of genomes.

### L1 retrotransposition occurs at very low level in the majority of tested adult stem cell types

Is L1 active in other somatic tissues besides the brain, or can all adult somatic stem cell types of the human body support L1 retrotransposition? In order to address this question, Macia and colleagues compared L1 expression and engineered L1 retrotransposition among various human cell types using isogenic cells [99]. To this end, the differentiation capability of hESCs was exploited to produce relatively pure populations of adult stem cells, which were then used to study L1 expression and retrotransposition. Notably, this approach allowed comparing different cell types that contain the same genetic background. Thus, L1 expression and retrotransposition was analyzed in keratinocytes (KERs, considered multipotent embryonic progenitor cells generating epidermal barrier, hair and nails), NPCs, fully differentiated neurons, MSCs and HSCs (Fig. 3). This allowed comparing NPCs and neurons to another ectoderm-derived cell type (KERs) and to multipotent stem cell types derived from mesoderm (MSCs and HSCs). Remarkably, data revealed that all cell types tested express endogenous L1-mRNAs and encoded L1-ORF1p, although there were significant differences in their expression levels (Fig. 3): L1-RNAs and L1-ORF1p were expressed at similar low levels in all cell types but NPCs, which expressed moderate levels of gene products as revealed by RT-qPCR, immunoblot analysis and confocal microscopy [99]. Consistently, the absence of detectable full-length L1 transcripts from human bone marrow-derived MSCs was demonstrated [158]. Analysis of the DNA-methylation status of L1 promoters in the various differentiated cell types tested uncovered an inverse correlation with L1 expression levels [99], which is consistent with the hypothesis that DNA methylation is a major mechanism to inhibit L1 retrotransposition in somatic cells [159].

To investigate whether KERs, HFFs, neurons and multipotent NPCs, MSCs and HSCs support L1 retrotransposition, Macia and colleagues took advantage of a hybrid adenovirus-based L1 vector that was specifically designed to conduct experiments in non-dividing cells [160]. Consistent with L1 expression data, L1 retrotransposition in all cell types tested, was extremely low, except for NPCs (Fig. 3). However, this study confirmed elevated retrotransposition levels in NPCs, and demonstrated that non-dividing mature neurons support L1 retrotransposition using engineered L1 reporter elements in vitro. Thus, this data suggests that L1 activity is not a generic property of all somatic stem cell types of the human body. Intriguingly, a recent report using transgenic mice carrying a human engineered L1 reporter element suggests that ionizing radiation does increase L1 expression in mouse HSCs, and that these cells might express higher levels of L1 RNAs than human HSCs [161]. Low levels of L1 retrotransposition observed in non-irradiated HSCs were also increased upon irradiation [161]. However, it could not be ruled out that some or most of the L1 retrotransposition events had accumulated already during embryonic development [161].

The above studies suggest that, among the cell types analyzed, L1 retrotransposition in adult multipotent stem cells occurs at a very low level, with the exception of NPCs (Fig. 3). These data also indicate that, even at low levels, L1 activity in multipotent stem cells would result in new L1 insertions carried by the derived specific tissues. Consistently, several putative somatic L1 insertions have been identified in healthy tissues, including liver [162], stomach [163] and esophagus [164]. Although in this report, one de novo insertion has been validated in tissues of colon [163] and esophagus [164], respectively, additional research is required to investigate to what extent endogenous L1 mobilization occurs in healthy adult tissues or at any earlier developmental stage. Considering that (just as neuronal cells) the gastrointestinal tract is derived from endoderm, extending L1 mobilization analyses to adult stem cells derived from this germ layer may clarify if the capability to support L1 retrotransposition is restricted to NPCs or also applies to endodermal stem cell populations. It was shown recently that heritable L1 insertions occur not only in pluripotent embryonic cells, but also in early primordial germ cells (PGCs), at least in mice [113]. In humans, PGCs represent the primary undifferentiated stem cell type that differentiates towards gametes (spermatozoa and oocytes). Due to the therapeutic potential of PGCs for the treatment of infertility [165], the impact of L1 activity in those cells should be given due regard. In summary, among the adult stem cell types tested to date, somatic L1 retrotransposition is restricted to NPCs and PGCs, and does not seem to be a generic property of multipotent adult stem cells.

## Regulation of TE activity in pluripotent stem cells

The human host is in a constant battle with TEs such as L1 to prevent their amplification and overreaching activity, because functional, active L1 elements carry substantial mutagenic potential. Somatic L1-mediated retrotransposition in somatic cells, such as stem cells, has also been linked to disease, especially in the context of cancer [75, 76, 162, 166]. Mobility restriction of these elements is therefore critical to maintain genome stability, especially during germline establishment and early embryogenesis, as new TE insertions in these cells can potentially be transmitted to the next generation [167].

During the first few days of embryogenesis, most TEs are targeted by silencing mechanisms and kept under control during early embryogenesis by epigenetic silencing and post-transcriptional regulation of L1 mRNAs [99, 168, 169]. Epigenetic silencing of TE expression is mediated by 1) Krüppel-associated box domain-containing zinc finger proteins (KRAB-ZFPs), mediators of heterochromatic formation [98] 2) cytosine methylation, a process governed by the action of DNA methyltransferase-3 like protein in germ cells [93], and 3) ten-eleven translocation (TET) family proteins [170]. TEs are recognized by sequence-specific proteins- or RNA-based repressors, with subsequent recruitment of heterochromatin-inducing complexes [171]. Histone methylation, histone deacetylation, and DNA methylation control TE expression and repress their *cis*-acting transcriptional components, which would otherwise activate neighboring genes via promoter or enhancer effects [172–178]. Histone repressive marks play a major role in this process, and trimethylation of histone 3 at lysine 9 (H3K9me3) is a modification found in a wide variety of TEs, including ERVs, LINEs, SINEs, and SVAs in hESCs [98, 179–184]. Excellent reviews have been published recently on how the host controls the activity of active TEs [67, 110, 155]. In the following section, we will address those host-encoded mechanisms that were reported to interfere with the retrotransposition cycle specifically in pluripotent stem cells.

### Restriction of TE transcription

**KRAB-ZFPs** are the largest family of transcriptional regulators in higher vertebrates [185] and play a major role in the early embryonic control of vertebrate TE expression, including ERV, LINE, SINE and SVA elements [98, 178, 184]. Harbouring an N-terminal KRAB domain and a C-terminal array of DNA-binding zinc fingers, they mediate silencing by recruiting the cofactor KRAB-associated protein 1 (KAP1, also known as TRIM28), which acts as a docking protein for proteins with heterochromatin-forming activities, including the H3K9 methyltransferase SETDB1 [178, 186]. The KRAB/KAP1 system represses transcription of endogenous retroelements primarily via histone deacetylation, H3K9 trimethylation, and HP1 recruitment, with DNA methylation occurring only secondarily to ensure the permanence of the silencing process [187, 188].

Evolutionary old L1 subfamilies that are between ~ 7 and 25 million years of age are silenced in hESCs [98, 185, 189] through specific KRAB-ZFPs such as ZNF93/KAP1, and there is evidence suggesting that KRAB-ZFPs also bind the younger and retrotransposition-competent L1Hs elements [185]. Beside L1s, in hESCs two-thirds of endogenous retroelements bound by KAP1 are SVAs, suggesting KAP1 is a major repressor of SVA activity. In summary, it is now well established that KRAB-ZFP/KAP1 complexes control TE activity in hESCs, including retrotransposition-competent elements harbouring the potential to affect genomic integrity, and help maintaining transcriptional homeostasis and normal differentiation of ESCs.

**DNA-Methylation** at CpG dinucleotides plays a pivotal role in TE silencing in both somatic and germ cells, and has been suggested to have evolved primarily to protect the host against TEs. [93, 190, 191]. In mammals, CpG methylation is established by DNA methyltransferases DNMT3a, 3b, 3L and 3C and maintained by DNA methyltransferase DNMT1 and cofactors. DNMTs are required to reestablish TE DNA methylation after the two waves of reprogramming undergone by germ cells and early embryos [192–195]. DNA-methylation is known to control L1 expression, and the L1 promoter has a canonical CpG island whose methylation is inversely correlated with L1 expression [93, 148, 149, 196].

As early embryonic models, hESCs/hiPSCs show significant hypomethylation of L1 promoters, overexpress full-length L1 mRNA, L1 ORF1p and L1-RNPs [79, 96–101, 132, 133, 197, 198]. However, DNA methylation is also involved in the long-term transcriptional control of currently inactive L1 s, and in hESCs, L1PA4 and L1PA5 exhibit higher methylation levels than the currently active L1Hs elements [98]. Investigation of the correlation between CpG methylation and TE expression in naïve and primed human ESCs uncovered that TEs in naïve cells were hypomethylated relative to primed cells [128]. Despite the overall low methylation in naïve hESCs, endogenous SVAs, which represent the most overexpressed TE family in these cells, tended to be more hypomethylated compared to non-overexpressed copies in naïve cells [128]. Thus, DNA methylation is key to control L1 expression and mobilization, and is the major TE controlling mechanism during gastrulation [125].

**TET proteins**: While the primary mechanism underlying DNA demethylation during the early embryonic period is replication-coupled dilution of 5mC [199, 200], an active mechanism dependent on TET enzymes has recently been uncovered to erase DNA methylation at specific loci in this period [170, 201–203]. TET enzymes catalyze the oxidation of 5-methylcytosine to 5-hydroxymethylcytosine (5hmC), and further to 5-formylcytosine (5fC) and 5-carboxylcytosine (5caC), which can be replaced with unmodified cytosine by base excision repair [204, 205]. Some of the TET proteins are highly expressed in ESCs and blastocysts [206], and it was reported that TET binding and demethylation at particular TE classes, such as LTR retrotransposons, acts in concert with pluripotency factors Nanog, Oct4 and Sox2 to maintain expression of a subset of genes in ESCs [170]. Depletion of TET1 and TET2 in mESCs has been shown to cause loss of 5hmC in the 5′ region of L1 [207]. In hESCs, TET proteins were shown to preferentially bind to evolutionary young, functional L1 elements, and participate in their active demethylation, but do not interact with older, inactive subfamilies. Although TETs drive L1 demethylation, L1s can be kept repressed through the TET-dependent recruitment of the transcriptional repressor SIN3A. The SIN3A co-repressive complex binds functional L1s in mouse and human ESCs ensuring their repression in a TET-dependent manner [92, 170]. Thus, and instead of being only positive regulators of L1 expression, TET enzymes may have a dual role in TE regulation by also recruiting SIN3A to demethylated L1 elements. A recent report provided evidence that the methyl-CpG binding domain as well as the adjacent non-sequence specific DNA binding domain of MeCP2 mediate repression of TET1-induced L1 mobilization [208]. Also, the KZFP/KAP1 complex was recently reported to maintain heterochromatin and DNA methylation at TEs in naïve murine ESCs partly by protecting these loci from TET-mediated demethylation [209].

**Small RNAs** classes include PIWI-interacting RNAs (piRNAs), endogenously produced small interfering RNAs (siRNAs) or micro RNAs (miRNAs), and the importance of small RNA-based repression in the control of L1 expression in human pluripotent stem cells has been reported and reviewed [114, 210, 211]. Double-stranded RNAs (dsRNAs) with sequence specificity to portions of the transcriptome that originate from TEs with bidirectional promoters, such as L1s, was suggested to serve as substrate for the production of siRNAs [212]. Moreover, dsRNA produced from the 5’UTR of L1 may trigger the interferon-dependent restriction factor ribonuclease L in some cancer cells, leading to L1 mRNA degradation [213].

**piRNAs** are a complex class of small non-coding RNAs that comprise mostly 24–32 nucleotides, specifically interact with the PIWI protein subfamily of the ARGONAUTE family [214], and act to repress mobile genetic elements in the germline of *Drosophila* and mammals [169]. piRNAs are generated by transcription of long TE clusters, resulting in the accumulation of short mature piRNAs in the cytoplasm by the ping-pong mechanism [215]. piRNAs then act as guides to destroy complementary TE transcripts by endonucleolytic cleavage. PIWI-mediated control is indeed triggered by the recognition of L1-proximal sequences by a complex encompassing a member of the PIWI subclade of Argonaute proteins and L1-derived piRNAs, which leads to L1 transcriptional inhibition via DNA methylation [216–218]. The piRNAs–PIWI system and DNA methyltransferases, acting downstream of piRNA action, play a crucial role in the early embryonic control of the youngest and mobile L1 lineages in human pluripotent cells [98, 186, 211, 219].

A role for miRNAs and the miRNA biogenesis machinery in controlling human non-LTR retrotransposons has been suggested by demonstrating that a complex termed Microprocessor, which comprises the RNase III type enzyme Drosha and its partner DGCR8, catalyzes the nuclear step of microRNA biogenesis [220, 221] and binds L1, *Alu* and SVA-derived small RNAs in human cells [210]. The results suggest that Microprocessor recognizes and processes structural regions within retrotransposition-competent L1 and *Alu* elements leading to a decrease of functional L1 and *Alu* RNAs which could result in a reduction of L1 and *Alu* retrotransposition frequencies [169, 210]. miR-128, a member of the class of miRNAs encompassing 20- to 24-nt-long noncoding RNAs that inhibit translational initiation and stimulate decay of mRNA targets [222, 223], was recently reported to decrease both amounts of full-length L1 RNA and engineered L1 retrotransposition frequencies in a cell culture based assay in human tumor cells and induced pluripotent stem cells [198].

### Post-transcriptional control of TEs

#### APOBEC3 proteins

The human APOBEC3 (Apolipoprotein B mRNA Editing Enzyme Catalytic Polypeptide 3, A3) protein family of cytidine deaminases comprises seven members (APOBEC3A [A3A], APOBEC3B [A3B], APOBEC3C [A3C], APOBEC3DE [A3DE], APOBEC3F [A3F], APOBEC3G [A3G], APOBEC3H [A3H]) [224–227], that inhibit the human non-LTR retrotransposons L1 and *Alu* with varying degrees of efficiency [reviewed in [228–230]. While A3B, A3C, A3DE, A3F and A3G were shown to be expressed in hESCs, A3A is absent from hESCs [105], but transiently overexpressed by up to 10-fold during reprogramming [132]. A3B is a nuclear protein that is also expressed in hiPSCs [105, 231, 232] and was demonstrated to restrict L1 retrotransposition in both hESCs [105] and hiPSCs in a deaminase-independent manner [211]. While it is likely that A3A inhibits L1 retrotransposition by deaminating transiently exposed L1 DNA [233], both A3C and A3DE were reported to restrict L1 retrotransposition through editing-independent mechanisms by interacting with ORF1p, thereby interfering with L1 RT activity [234, 235]. Additional mechanistic studies are required, because contradictory results were found among cell lines and studies.

**SAMHD1**, a dGTP-activated deoxynucleoside triphosphate triphosphohydrolase that inhibits L1 retrotransposition in actively dividing cells by reducing the L1 ORF2p level [236], was demonstrated to be transiently induced during the initial 10 days of reprogramming [132], and could therefore play a role in the restriction of L1 mobilization during this period. In addition to diminishing L1 reverse transcription by reducing L1 ORF2p expression [236], SAMHD1 was recently reported to stimulate the formation of stress granules and thus enhance the sequestration of L1 RNP complexes in these granules [237].

**TEX19.1** is expressed in germ cells, pluripotent cells and the placenta of mammals [238] and was recently shown to regulate L1-ORF1p levels and mobilization of engineered L1 elements in pluripotent mouse embryonic stem cells [106]. Mouse TEX19.1, and its human ortholog TEX19, physically interact with L1-ORF1p, and can regulate L1-ORF1p abundance at postranslational level through stimulating its polyubiquitylation and proteasome-dependent degradation [106].

For the sake of completeness, we also mention the following host-encoded factors which also restrict L1 retrotranspositrion mostly by post-transcriptional mechanisms, but have not been reported to restrict L1 mobilization in stem cells so far: (i) MOV10, an RNA helicase that mediates access of the RNA-induced silencing complex to messenger RNAs [239, 240] (ii) ZAP, an antiviral member of the poly (ADP-ribose) polymerase (PARP) protein family [241, 242] (iii) TREX-1, a three-prime DNA exonuclease [243, 244].

## DNA transposons as tools for genetic engineering of stem cells

**Fig. 4:**
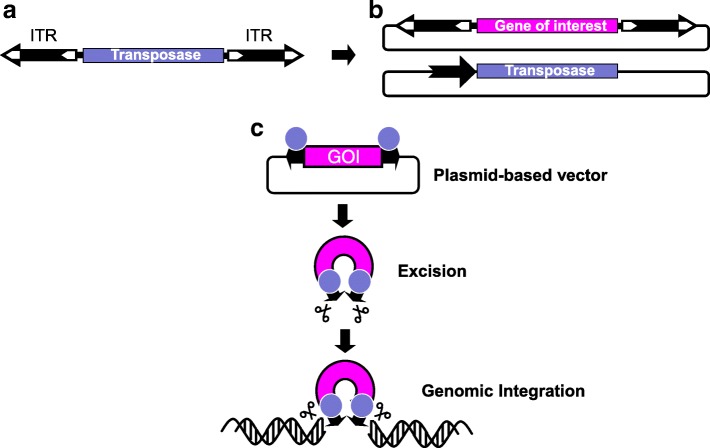
Use of class II transposons as gene vectors. **a** Autonomous DNA transposons consist of a transposase-coding gene that is flanked by inverted terminal repeats (ITR; black arrows flanked by white arrows). **b** Bi-component transposon vector system for delivering plasmid-encoded transgenes. One component consists of a plasmid containing a gene of interest (GOI) flanked by transposon ITRs. The second component is a transposase expression plasmid. Black arrow, promoter driving expression of transposase gene. **c** The transposon carrying a GOI is excised from the donor plasmid and integrated at a chromosomal site by the transposase

An important point of intersection between stem cells and transposable elements is genetic engineering for the purposes of deciphering disease mechanisms and for gene- and cell-based therapeutics. DNA (Class 2) transposons (Fig. 4a) became major tools for genome manipulations in a wide range of cell types, including stem cells (reviewed by [245–247]. The mobility of DNA transposons invariably depends on two functional components: a protein component called the transposase and a DNA component called inverted terminal repeats (ITRs) supporting binding of and cleavage by the transposase (Fig. 4a). Due to their attributes including efficient gene transfer in human cell types, ease of experimental manipulation, and detailed understanding of the transposition process, the *Sleeping Beauty*, *piggyBac* and *Tol2* transposons have become the transposons of choice for stem cell engineering. What is common to all gene transfer applications by any transposon is the setup of a conditional, two-component gene delivery system, in which a gene of interest flanked by the transposon ITRs is mobilized out of standard plasmid vectors by the transposase that is conditionally and transiently expressed in the relevant cells (Fig. 4b and c). Stem cells are ideal targets for gene therapy applications. It is of great hope to consider stem cells to achieve tissue repair or to restore and replenish cells in the background of a genetic disease. Following a transposon-based genetic manipulation step to introduce a gene of interest, such as a therapeutic gene rendering stable phenotypic correction, stem cells can be expanded in vitro and then subjected to differentiation into particular cell lineages according to the specific therapeutic need. There is now widespread evidence for robust transposon-mediated gene transfer in several, clinically relevant stem cell types, such as ESCs, iPSCs, CD34+ hematopoietic stem cells (HSC), MSCs, or myoblasts.

### Transgene expression in hESCs by means of the *Sleeping Beauty* transposon system

A proof of concept study established efficient and stable transgene expression in hESCs using the *Sleeping Beauty* transposon system. In this study, transposons, carrying different transgenes coding for genetic markers, were effectively delivered to undifferentiated hESCs. The source of transposase was either DNA or mRNA. Molecular analysis indicated that 98% of stable gene transfer resulted from transposition events. These genetically engineered hESCs were then differentiated into teratomas in vivo and mature hematopoietic cells in vitro and the progeny maintained stable transgene expression [248]. Similarly successful gene transfer in hESCs was obtained with the *piggyBac* system as well, where fluorescent reporters were introduced into ESCs, and these remained functional following in vitro differentiaton. The *piggyBac* transposon system also allowed for seamless restoration of the insertion site following transposon removal by a second round of transient transposase expression [249]. The *Sleeping Beauty* transposon was also used to introduce a CAG promoter-driven GFP expression cassette to hESCs. During differentiation experiments the CAG promoter yielded outstanding GFP expression in cardiomyocytes allowing for specific labeling of cardiomyocytes in a spontaneously differentiated, mixed cell population [250]. The same transposon was also used to introduce a genetically encoded Ca^2+^ions indicator (GCaMP2) into hESCs allowing for real-time Ca imaging without the need for dyes. Cardiomyocytes differentiated from these ESCs were characterized by their spontaneous contractions and Ca^2+^ signal oscillations, presenting a powerful tool for pharmacological screening assays [251].

### Multiple applications of transposon systems in the hiPSC field

During the early developments in the iPSC field, a significant step was achieved when non-viral methods were used to achieve reprogramming. Both *piggyBac* [252] and *Sleeping Beauty* [253, 254] were successfully used to generate hiPSCs, and transposons remained relevant gene transfer tools in the context of hiPSCs generated from patient-derived cells with a disease-causing genetic background. In this scenario transposons can be effectively used to correct a disease phenotype by introducing functional genes into hiPSCs followed by differentiating the relevant cell types that can be used for therapeutic purposes. In a preclinical mouse model of Duchenne muscular dystrophy, hiPSCs were corrected with the *Sleeping Beauty* transposon system carrying the micro-utrophin gene. Cells were then differentiated into skeletal muscle progenitors. Following transplantation of these progenitors into dystrophic mice, engrafted muscles displayed large numbers of micro-utrophin-positive myofibers, with biochemically restored dystrophin-glycoprotein complex and improved contractile strength [255]. Transposon-based gene delivery is not only useful for correcting a genetic disease, but can also be applied to coax differentiation into a particular cell lineage. For example, drug-inducible expression of MYOD1 from a *piggyBac* transposon vector in hiPSCs for at least 5 days was demonstrated to lead to highly efficient differentiation into myocytes [256]. In a similar experiment, *Sleeping Beauty*-mediated overexpression of PAX3 in iPSCs induced differentiation into MYOD positive myogenic progenitors and multinucleated myofibers [257]. Transposons can also be used in combination with designer nucleases in iPSCs to correct gene defects. For example, the efficiency of endonuclease-based gene targeting can be enhanced by using the *piggyBac* transposon as an efficient, transient drug selection tool due to the possibility of seamless removal of the drug marker enabled by the re-transfection of the transposase [258]. In ß-thalassemia patient-derived iPSCs, *piggyBac* transposon-mediated puro∆tk-based drug selection was used in combination with CRISPR/Cas9 to achieve correction of the mutation in the Hemoglobin Beta Chain (HBB) gene [259]. A similar strategy was used in combination with CRISPR/Cas9 in Huntington disease iPSCs to correct mutations in the Huntigtin (HTT) gene. The corrected cells were then successfully differentiated into excitable, synaptically active forebrain neurons [260]. A different combination of CRISPR/Cas9 and *piggyBac* transposon demonstrated the versatility of combinations with different genome editing tools in iPSCs. The Cas9 gene driven by an inducible promoter was delivered by the transposon, and genomic modification was achieved following sgRNA delivery. Following a transient transposase expression the inducible Cas9 cassette was removed yielding a genome-edited iPSC clone with seamless transgene removal [261].

### Efficient gene transfer into mobilized HSCs and human CD34^+^ cord blood cells

A rapidly progressing area for transposon-mediated gene therapy applications is represented by HSCs. Transposon-based tools hold great promise for rendering efficient gene correction without the potential risks inherent to viral delivery methods, which resulted in severe adverse reactions in clinical trials in the past [262, 263]. HSCs have the potential for self-renewal and maintenance of the ability to differentiate into hematopoietic lineages, and are thus ideal targets for gene therapy applications in hematologic diseases. HSCs can be efficiently modified by the *Sleeping Beauty* and *piggyBac* transposon systems [264]. Especially, developments of *Sleeping Beauty* transposons were taking shape in recent years enabled by the highly efficient SB100X hyperactive transposase in CD34^+^ HSCs [265]. The *Sleeping Beauty* transposon system not only supports the efficient gene transfer into mobilized CD34^+^ HSCs, but also into human cord blood CD34^+^ cells as shown in a model of sickle cell disease [266]. Further developments of the transposon-mediated delivery were also undertaken in recent years to target CD34^+^ HSCs. For example, an interesting and promising optimization step was undertaken when the transposon was delivered to the cells as a minicircle in combination with transposase supplied as mRNA. This approach led to improved cell survival and reduced cytotoxicity in the HSCs providing also several biosafety advantages over conventional delivery methods [267]. The combination of the *Sleeping Beauty* transposon system and adenoviral vectors in a hybrid vector system was also proposed as a promising method to achieve in vivo gene delivery. This approach holds broader clinical application potential for gene therapy as it may circumvent the need for myeloablation and transplantation. In this study HSCs were mobilized into peripheral blood in a transgenic, humanized mouse model, and were targeted using a hybrid adenovirus/transposon vector system injected intravenously in vivo, resulting in functional HSCs homing back to the bone marrow stably expressing the transgene [268].

MSCs are also in the focus of regenerative medicine, however, there are no breakthrough applications yet, and the field is still hampered by many controversies. Nevertheless, it has been evidenced that both *Sleeping Beauty* and *piggyBac* transposon-based gene transfer is applicable in MSCs as well. The genetically engineered MSCs are still characterized by the ability to undergo osteogenic, myogenic, and adipogenic differentiation after modifications with the transposons [257, 269]. *piggyBac*-mediated gene transfer of IFNγ into adipose-derived MSCs was used in a mouse model of melanoma to show that the IFNγ-expressing MSCs engrafted into tumor stroma, inhibited tumor growth and angiogenesis, and prolonged the survival of mice [270].

Myoblasts are self-renewing adult muscle progenitor cells, which differentiate into skeletal muscle cells and could potentially be harnessed for cell therapy of muscle disorders. Both *Sleeping Beauty-* and *piggyBac*-mediated gene transfer methods can be applied to efficiently modify myoblasts and were shown to be useful tools in delivering therapeutic genes into myoblasts. Proper dysferlin expression as well as highly efficient engraftment of the engineered myoblasts was evidenced in the skeletal muscle of dysferlin-deficient mice [271], whereas transposon vectors encoding microdystrophins and delivered into myoblasts have been shown to yield proper expression levels in differentiated multinucleated myotubes [272].

### Insertional mutagenesis and genetic labeling studies

DNA transposons have also been successfully used in stem cells to identify different mechanisms of stemness and differentiation in insertional mutagenesis and genetic labeling studies. For such applications, the transposon vectors are equipped with mutagenic and reporting features (e. g., gene traps) that allow conditional expression of a marker upon integration into a gene (reviewed in [246]. For example, a modified *Sleeping Beauty* transposon was generated to randomly trap genes in the neural stem cell genome and modify their expression or tag them with fluorescent markers and selectable genes, allowing recombinant cells to be isolated and expanded clonally. This approach may facilitate the identification of novel determinants of stem cell biology and neural cell fate specification in NSCs [273]. A modified *Tol2* transposon was also designed to allow for conditional disruption of a broad spectrum of genes. The system was used in mouse ESCs, and relied on differentially tagged *Tol2* transposons to discern individual integrations within a single cell [274]. A *piggyBac* transposon-based insertional mutagenesis method has been developed to efficiently generate genome-wide mutant libraries in mouse haploid ESCs [275]. In hESCs a *piggyBac* insertional mutagenesis screen was used to identify the role of nuclear RhoA during stem cell differentiation [276]. The *Sleeping Beauty* transposon has been also used to explore the clonal dynamics of native haematopoiesis in vivo allowing for a specific fate tracking approach based on in situ labelling of HSCs. DsRed-positive HSCs harboring distinct insertion sites resulted from inducible transposition events fully reconstituted myeloid and lymphoid lineages evidencing successful tagging. These experiments established that steady-state blood production is maintained by the successive recruitment of thousands of clones, each with a minimal contribution to mature progeny. These results demonstrated that large numbers of long-lived progenitors are the main drivers of steady-state haematopoiesis during most of adulthood [277].

### The risk of genotoxicity caused by DNA transposon activity

As discussed above in the context of endogenous TEs, one of the most important risk factors also associated with the use of transposon-based gene transfer tools in stem cells is genotoxicity. In the context of a transposon vector system, at least two fundamental properties can contribute to genotoxicity: i) interaction of the transposase with endogenous human DNA sequences or human proteins with the transposon vector sequences and ii) the genome-wide insertion profile of the vector. With respect to “off-target” cleavage of the transposase, the use of the *Sleeping Beauty* and *Tol2* systems appears to be safe in human cells. Both of these transposons originate from fish genomes, and the mammalian lineage does not contain transposons sufficiently similar to allow cleavage by these transposases. Vice versa, human cells do not express proteins that could re-mobilize a genomically integrated *Sleeping Beauty* or *Tol2* vector. In contrast, the human PGBD5 transposase-derived protein was reported to mobilize *piggyBac* transposon vectors in human cells [278], thereby presenting potential implications for human applications [279].

Characterization of the target site selection properties of different vector systems is highly useful for ranking the different vector types and designs according to their genotoxic potential [280]. The *Sleeping Beauty* transposon displays the least deviation from random with respect to genome-wide distribution: no apparent bias was seen for either heterochromatin marks or euchromatin marks and only a weak correlation with transcriptional status of targeted genes was detected [281]. This is in marked contrast to target site distributions of several other transposons including *Tol2* [264, 282], and *piggyBac* [264, 283, 284] that favor integration into genes and near chromatin marks characteristic of active transcription units (e.g., H3K27 acetylation and H3K4 monomethylation). The *piggyBac* transposon, in particular, has been shown to favor open chromatin, expressed genes and TSSs (±5 kb) associated with DNaseI hypersensitive sites, H3K4Me3 marks and Pol II-bound regions in mouse and human cells [283–288]. These observations collectively suggest that *Sleeping Beauty* might be the safest currently available transposon for therapeutic gene delivery in clinical trials.

## Conclusions

Stem cell therapies have been expected to bring substantial benefit to patients suffering a wide range of diseases and injuries. It was expected that the benefits of bone marrow transplants for patients needing reconstruction of their hematopoietic and immune system would apply to stem cell transplants of other cell types, and optimism has been high for the utilization of embryonic and induced pluripotent stem cells for a variety of applications. However, before these promising stem cells or their differentiated derivatives are administered to patients, genomic integrity of these cells has to be ensured to guarantee that these cells remain therapeutically functional and are not tumorigenic. In recent years, a remarkable amount of data accumulated showing that the activity of endogenous TEs can be one source of genomic destabilization in stem cells, and constitutes a risk for the biosafety of stem cell-based therapies. By giving an overview of the potentially mutagenic activity of TEs in human multipotent and pluripotent stem cells, the consequences of their activity for the genomic integrity and host gene expression, we provide arguments for a thorough characterization of TE activity and its consequences in the individual stem cell lines before their therapeutic utilization in patients in order to ensure biosafety of these stem cells cells and/or their applied derivatives.
